# Development of an imaging based virus aggregation assay for vaccine development

**DOI:** 10.1186/1742-4690-9-S2-P319

**Published:** 2012-09-13

**Authors:** D Stieh, C Gioia, M McRaven, G Cianci, P Kiser, T Hope

**Affiliations:** 1 Northwestern University, Chicago, IL, USA; 2 University of Utah, Salt Lake City, UT, USA

## Background

Vaccination strategies capable of eliciting neutralizing antibody responses to HIV remain elusive despite extensive efforts. Alternative antibody functions offer opportunities for protection without necessarily achieving broad neutralization breadth. Viral immune exclusion through aggregation has been proposed as an alternative protection pathway, but mechanisms for studying this phenomenon at the scale necessary for clinical trials have not been explored.

## Methods

Concentrated fluorescent virions of two colors, suspended in hydroxyethylcellulose (HEC) gel, which has been formulated to simulate the diffusion characteristics of cervical mucus, are imaged over time. Mean squared displacement and incidence of colocalized viral particles are determined. The addition of monoclonal antibodies of various specificities and isotypes affects these parameters is explored. Immunoglobulin isolated from HIV-1 positive individuals was examined as well. Correlative scanning electron micrographs of the same preparations were performed to confirm the nature of suspected aggregates.

## Results

Multimeric antibodies, rather than monomeric isoforms, selectively hinder the diffusion characteristics of colocalized virions, more so than non-aggregated virions. The incidence of colocalized virions is also increased in a concentration dependent manner. Excessive antibody or virus concentration is seen to obviate aggregate formation. These results are seen in both monoclonal antibody experiments alongside polyclonal patient IgA isolated from breast milk and IgM from serum.

## Conclusion

Virus aggregation has been demonstrated as a feasible effector function of HIV-1 specific antibody preparations. This assay platform is amenable to adaptation for medium to high throughput sample screening and low sample size. Monoclonal antibodies as well as patient samples are able to induce similar behaviors. Measurement of viral aggregation as a corollary of vaccine efficacy is deserving of further exploration in a clinical setting.

